# Disseminated Histoplasmosis Presenting as an Isolated Oral Ulcer in a 91-Year-Old Patient Without Recognized Immunodeficiency: A Case Report

**DOI:** 10.7759/cureus.111142

**Published:** 2026-06-19

**Authors:** Muhammad Usman, Laiba Murtaza, Muhammad Zawar Asif, Muhammad Usman Shahbaz, Hudaib Ansari

**Affiliations:** 1 Internal Medicine, Mercy Hospital Fort Smith, Fort Smith, USA; 2 Internal Medicine, Arkansas College of Osteopathic Medicine, Fort Smith, USA; 3 Internal Medicine, King Edward Medical University, Lahore, PAK

**Keywords:** adrenal histoplasmosis, clinical case report, disseminated histoplasmosis, frail elderly, histoplasma in immunocompetent patient, immunosenescence, oral ulcer, progressive disseminated histoplasmosis

## Abstract

Disseminated histoplasmosis is commonly seen in immunocompromised individuals with acquired immune deficiency syndrome (AIDS). It is relatively uncommon in individuals without recognized immunodeficiency and typically affects older men. We present a case of a 91-year-old male with no apparent immune deficiency disorder and having a remote history of occupational exposure to chicken houses who presented with an isolated, painful left buccal cavity ulcer, which was initially diagnosed as an aphthous ulcer. After six weeks of progressive symptoms and poor nutritional intake, the biopsy of the ulcer revealed mucocutaneous histoplasmosis. During that time, the patient also developed cytopenias, predominantly leukopenia and thrombocytopenia. Abdominal imaging revealed bilateral adrenal masses. Bone marrow biopsy, as well as adrenal biopsy and culture, were consistent with disseminated histoplasmosis. Due to extreme frailty and poor nutritional intake, amphotericin B was deferred. Administration of itraconazole also became challenging due to severe oral ulcer pain, and eventually, a percutaneous endoscopic gastrostomy (PEG) tube was placed. Despite adequate antifungal therapy via PEG tube, the patient continued to remain extremely frail and ultimately died after transitioning to hospice care within one week of diagnosis of disseminated histoplasmosis. This case illustrates how an isolated oral ulcer can be the initial and dominant clinical manifestation of widespread disseminated histoplasmosis likely related to underlying immunosenescence. It also highlights a critical gap in the treatment guidelines for disseminated histoplasmosis in an extremely elderly and frail population.

## Introduction

*Histoplasma capsulatum* is a dimorphic fungus that causes histoplasmosis and is endemic to the Mississippi and Ohio River Valley. It is commonly acquired through inhalation from soil enriched with bird or bat droppings [1]. The primary infection in individuals without recognized immunodeficiency is usually self-limited; however, the organism itself can stay dormant and reactivate years to decades later as T-cell-mediated immunity fades, similar to other latent granulomatous infections [2]. Disseminated histoplasmosis classically manifests as febrile illness, night sweats, generalized fatigue, weight loss, pancytopenia, and painful oral ulcers, and is more common in the elderly population [1]. Due to its variable presentation in combination with low clinical suspicion in patients without recognized immunodeficiency, disseminated histoplasmosis contributes to significant diagnostic delays. The median time from symptom onset to diagnosis is reportedly longer in the population without recognized immunodeficiency compared to the immunocompromised population with fewer clinical, laboratory, and radiographic features present at the time of diagnosis [3]. A retrospective cohort of 347 patients reported significantly high all-cause mortality at six months in the older population, with the older population less likely to present with typical features of hilar lymphadenopathy and chest pain, thus leading to diagnostic complexity [4]. Current treatment guidelines recommend liposomal amphotericin B induction therapy for moderate-to-severe disseminated histoplasmosis; however, these recommendations are primarily derived from younger, HIV-positive populations. The guidelines lack specific recommendations for extremely elderly and frail populations where amphotericin B toxicity may be prohibitive [1]. 

We report a case of fatal disseminated histoplasmosis in a 91-year-old male without recognized immunodeficiency who presented with an isolated painful oral ulcer as the initial and dominant clinical manifestation, followed by progressive functional decline. Subsequent workup revealed clinically silent disseminated histoplasmosis to bone marrow and bilateral adrenal glands. This case highlights the possible role of age-related immune system weakness in the reactivation of latent infection from prior occupational exposure. It also exposes a critical treatment gap in select populations where the risks of standard induction therapy may outweigh potential benefits.

## Case presentation

Our patient is a 91-year-old male with a medical history significant for essential hypertension, gastroesophageal reflux disease, chronic obstructive pulmonary disease, and remote tobacco use, who presented to the hospital with generalized fatigue, weight loss, poor nutritional intake, and a progressively painful oral ulcer. The ulcer first appeared approximately six weeks ago as a sore spot on the left side of his oral cavity. The patient reported that he used to work in chicken houses before retiring at age 70, approximately 21 years prior. His dentist evaluated the lesion approximately four weeks prior and diagnosed it as an aphthous ulcer with recommendations for supportive care. The patient denied any systemic symptoms--no fevers, night sweats, cough, dyspnea, orthopnea, nausea, or vomiting--making the oral ulcer his initial and dominant clinical complaint. Over the week preceding the current presentation, ulcer pain had worsened to the point of complete inability to eat, thus resulting in severe generalized weakness and failure to thrive. 

Physical examination revealed a frail elderly male with a left buccal ulcer with nodular and irregular surface and raised borders without any bleeding; the remainder of the physical examination was unremarkable.

At the time of presentation, his lab work revealed leukopenia, thrombocytopenia, and mild anemia. Of note, his lab work about six weeks prior was unremarkable for any of the cytopenias. The initial laboratory evaluation is summarized in Table 1.

**Table 1:** Initial laboratory results.

Test name	Value	Reference range
White blood cells	2.7 K/uL	4.2-9.1 K/uL
Red blood cells	4.66 M/uL	4.63-6.08 M/uL
Hemoglobin	13.4 g/dL	13.7-17.5 g/dL
Hematocrit	39.3%	40.1-51.0%
Mean corpuscular volume	84.3 fL	79.0-92.2 fL
Mean corpuscular hemoglobin	28.8 pg	25.7-32.2 pg
Mean corpuscular hemoglobin concentration	34.1 g/dL	32.3-36.5 g/dL
Red cell distribution width	15.1%	11.6-14.4%
Platelets	81 K/uL	163-337 K/uL
Neutrophils absolute	2.17 K/uL	1.05-6.10 K/uL
Lymphocytes absolute	0.21 K/uL	1.10-3.70 K/uL
Monocytes absolute	0.17 K/uL	0.20-0.80 K/uL
Eosinophils absolute	0.11 K/uL	0.00-0.50 K/uL
Basophils absolute	0.02 K/uL	0.00-0.08 K/uL
Immature granulocytes absolute	0.03 K/uL	0.00-0.31 K/uL
Sodium	132 mmol/L	136-145 mmol/L
Potassium	3.8 mmol/L	3.5-5.1 mmol/L
Chloride	96 mmol/L	98-107 mmol/L
Carbon dioxide	25 mmol/L	22-29 mmol/L
Blood urea nitrogen	25 mg/dL	8-23 mg/dL
Creatinine	0.79 mg/dL	0.67-1.17 mg/dL
Glucose	92 mg/dL	74-99 mg/dL
Total protein	6.2 g/dL	6.6-8.7 g/dL
Albumin	3.4 g/dL	3.5-5.2 g/dL
Total bilirubin	1.7 mg/dL	0.0-1.2 mg/dL
Alkaline phosphatase	94 U/L	40-129 U/L
Aspartate aminotransferase	23 U/L	0-50 U/L
Alanine aminotransferase	8 U/L	0-50 U/L
Glomerular filtration rate	>60 mL/min/1.73 m²	-
Urine pH	6.0	5.0-8.0
Leukocyte esterase	Negative	Negative
Nitrites	Negative	Negative
Urine protein	1+	Negative
Urine ketones	3+	Negative
White blood cells urine	0-2/hpf	0-2/hpf
Red blood cells urine	0-2/hpf	0-2/hpf
Bacteria	Negative	Negative/hpf

CT scan of facial bones and sinuses with contrast was unremarkable for any acute abnormality. The patient was ultimately evaluated by an otolaryngologist, and a biopsy of the oral ulcer was performed. The biopsy results showed acute squamous inflammation with underlying diffuse histiocytic inflammation and positive Grocott-Gomori's methenamine silver stain (GMS) for histoplasma (Figure 1).

**Figure 1:**
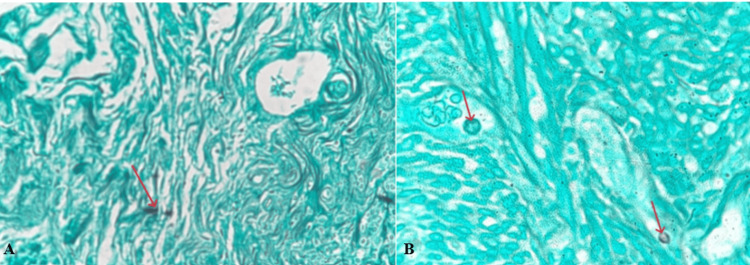
Grocott-Gomori methenamine silver (GMS) stain of the left buccal mucosal biopsy. (A) Low-power view (original magnification ×40) demonstrating diffuse histiocytic inflammation within the submucosal tissue with scattered dark-staining yeast forms (red arrow). (B) High-power view (original magnification ×100, oil immersion) revealing small (2-4 μm), round-to-oval, narrow-based budding yeast forms consistent with Histoplasma capsulatum (red arrow).

After the diagnosis of mucocutaneous histoplasmosis, the infectious disease specialist was consulted. Further lab work is summarized in Table 2.

**Table 2:** Infectious and immunologic testing. dsDNA: double-stranded deoxyribonucleic acid, RNP: ribonucleoprotein, SCL-70: scleroderma-70 (topoisomerase I). Bold values indicate abnormal results.

Test name		
Human immunodeficiency virus 1/2 antibodies and antigen	Non-reactive	Non-reactive
Cryptococcal antigen	Negative	Negative
Cytomegalovirus polymerase chain reaction	Negative	Negative
Gastrointestinal pathogen polymerase chain reaction panel	Negative	Negative
Blood culture	No growth	-
Urine culture	No growth	-
Blastomyces antigen quantitative	9.66 ng/mL	0.31 ng/mL-20.00 ng/mL
Antinuclear antibody titer	1:80	<1:40 negative; 1:40–1:80 low; >1:80 elevated
Antinuclear antibody pattern	Nuclear, homogeneous	-
Autoimmune panel (dsDNA, Smith, RNP, Sjogren, SCL-70, Centromere, Ribosomal P Protein, Rheumatoid Factor, Cyclic Citrullinated Peptide)	Negative	Negative
Beta-D-glucan	308 pg/mL	<60 pg/mL
Histoplasma urine antigen	5.8 ng/mL	<0.2 ng/mL

CT chest/abdomen/pelvis with contrast revealed heterogeneously enhancing bilateral adrenal masses (right: 4.1 × 2.5 × 3.9 cm; left: 3.9 × 3.3 × 5.7 cm) without pulmonary involvement or hepatosplenomegaly (Figure 2). 

**Figure 2:**
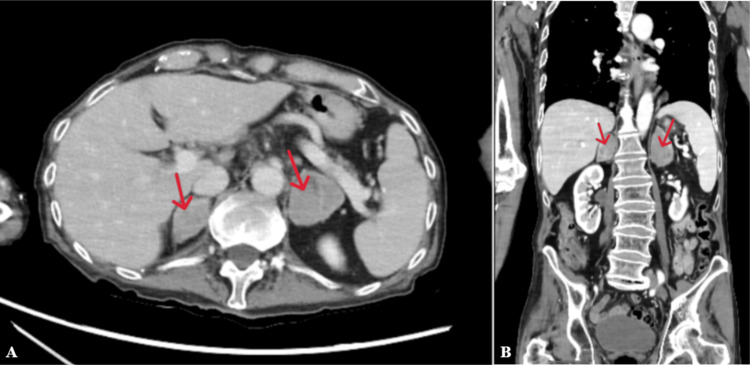
Contrast-enhanced computed tomography of the abdomen in axial (A) and coronal (B) planes demonstrating heterogeneously enhancing, indeterminate bilateral adrenal masses (red arrows). The right adrenal mass measures 4.1 × 2.5 × 3.9 cm, and the left adrenal mass measures 3.9 × 3.3 × 5.7 cm. Both masses appear as soft-tissue-density lesions with irregular heterogeneous enhancement, without evidence of local invasion or hepatosplenomegaly.

Bone marrow biopsy was performed with both core biopsy and aspirate smears staining positive for histoplasma on GMS stain; flow cytometry was negative for any myeloproliferative disorder. Left adrenal biopsy confirmed *Histoplasma capsulatum* on GMS stain and culture; acid-fast bacilli (AFB) stain was negative. Adrenal function testing (morning cortisol: 19 with reference range 4.6-20.6 mcg/dL, adrenocorticotropic hormone: 31 with reference range 9-52 pg/mL) was within normal limits with no clinical signs or symptoms of adrenal insufficiency. The final diagnosis of disseminated histoplasmosis involving oral mucosa, bone marrow, and bilateral adrenal glands was established, all unmasked by a single oral ulcer. 

During this time, the patient continued to deteriorate rapidly. Given his advanced age, severe frailty, poor functional reserve, and concern for treatment-related toxicity, liposomal amphotericin B was deferred after multidisciplinary discussion. As the oral ulcer precluded any oral intake, continuing itraconazole became challenging in our case. A percutaneous endoscopic gastrostomy (PEG) tube was placed after multidisciplinary discussion for nutrition and medication delivery. Drug monitoring confirmed therapeutic itraconazole levels (3.5 mcg/mL, reference value >2 mcg/mL).

The patient’s hospital course was further complicated by the development of abdominal distention requiring minimal-rate tube feeds, a small perioral abscess related to the oral ulcer that could not be drained, and progressive encephalopathy. Despite aggressive nutritional support and antifungal treatment with therapeutic antifungal levels, the patient continued to deteriorate rapidly.

After a goals-of-care discussion, the family opted for hospice care. The patient was admitted to the inpatient hospice unit and succumbed to the disease 72 hours later, approximately one week after the diagnosis of disseminated histoplasmosis was established.

## Discussion

This case highlights several important clinical aspects. An isolated painful oral ulcer served as the initial and dominant manifestation of widespread disseminated histoplasmosis and prompted the diagnosis of otherwise clinically occult disease. It also demonstrates that disseminated histoplasmosis may present without classic symptoms despite extensive organ involvement. Although oral ulcers are a recognized feature of progressive disseminated histoplasmosis, they typically occur alongside systemic symptoms such as febrile illness, night sweats, and weight loss [1]. 

The commonly reported age range for disseminated histoplasmosis in patients without recognized immunodeficiency is predominantly males in their 60s to 70s [1,4]; however, the patient's age of 91 years is well beyond the commonly reported range. 

In the Mayo Clinic 15-year institutional review of 111 patients with systemic histoplasmosis, the most frequently reported symptom (63%) was fever, followed by respiratory complaint (43%) and weight loss (37%); however, the patient lacked these common complaints despite extensive disease involvement [5].

The complete absence of systemic symptoms despite widespread involvement is highly unusual and may reflect decreased inflammatory response secondary to advanced immunosenescence, where decreased ability to mount systemic inflammatory response paradoxically masks the infection severity [2,6]. Due to its ability to mimic aphthous ulcers, squamous cell carcinoma, lymphoma, and tuberculosis, it has been called "the great imitator" [6].

The 21-year interval between last occupational exposure in chicken houses and disease presentation is consistent with the established capacity of histoplasma to remain dormant within the granulomas and reactivate as the ability of cellular immunity slowly decreases to contain these organisms [1,6].

Another notable finding was bilateral adrenal masses with preserved adrenal function. In a retrospective study, adrenal glands are affected in nearly 80% of patients with progressive disseminated histoplasmosis, and 41% of patients with adrenal histoplasmosis had primary adrenal insufficiency [7]. The preserved adrenal function in our patient, despite large bilateral masses with biopsy-proven histoplasmosis, suggests that sufficient functional adrenal reserve remained despite extensive adrenal involvement [7].

The absence of pulmonary findings on CT imaging further supports the mechanism of reactivation of latent disease instead of acute primary pulmonary disease, although this cannot be definitively established without further evaluation.

The most important aspect of our case was the therapeutic challenge. Liposomal amphotericin B at a dose of 3 mg/kg/day is recommended for induction in moderate-to-severe disseminated histoplasmosis, followed by stepdown to itraconazole [8]. In our patient, advanced age, severe frailty, and concern for treatment-related toxicity led to deferral of liposomal amphotericin B. Itraconazole was initiated; however, the oral ulcer and associated pain created substantial challenges for antifungal administration. Ultimately, a PEG tube was placed for nutritional support and medication delivery. 

A Mayo Clinic series of 10 patients with oropharyngeal histoplasmosis reported severe oral pain with ulcerations and a diagnostic delay in six of the patients due to various factors, including lack of biopsy or fungal staining [9]. A similar delay happened in our patient after an initial misdiagnosis as an aphthous ulcer.

Franklin et al. reported 21% 90-day mortality in disseminated histoplasmosis. These mortality rates, combined with the significant association of older age and mortality, highlight the poor prognosis of delayed diagnosis in extremely elderly patients with widespread disease [1,3]. 

The fulminant trajectory from a seemingly benign oral ulcer to death in a total of approximately seven weeks highlights the fatal consequences associated with delayed recognition of disseminated histoplasmosis despite therapeutic antifungal levels. It also emphasizes that alternate strategies or perhaps earlier diagnosis, enabling aggressive treatment with liposomal amphotericin B before advanced frailty, can lead to favorable outcomes in this population.

## Conclusions

This case demonstrates how an isolated oral ulcer can be the dominant manifestation of disseminated histoplasmosis in an elderly patient without recognized immunodeficiency, where clinically silent bone marrow and adrenal involvement were unmasked only after systemic workup. The fulminant clinical course from an isolated oral ulcer to death approximately seven weeks later underscores the potential severity of delayed diagnosis in disseminated histoplasmosis. The case also highlights diagnostic complexity when an oral ulcer is misdiagnosed as an aphthous ulcer, as well as a critical treatment gap for moderate-to-severe disseminated histoplasmosis in the elderly population, where amphotericin B induction therapy is not possible due to toxicity and frailty.
